# Understanding the Impact of 2D and 3D Fibroblast Cultures on In Vitro Breast Cancer Models

**DOI:** 10.1371/journal.pone.0076373

**Published:** 2013-10-04

**Authors:** Kyung Eun Sung, Xiaojing Su, Erwin Berthier, Carolyn Pehlke, Andreas Friedl, David J. Beebe

**Affiliations:** 1 Department of Biomedical Engineering, University of Wisconsin, Madison, Wisconsin, United States of America; 2 Department of Medical Microbiology, University of Wisconsin, Madison, Wisconsin, United States of America; 3 Department of Pathology and Laboratory Medicine, University of Wisconsin, Madison, Wisconsin, United States of America; 4 Paul P. Carbone Comprehensive Cancer Center, University of Wisconsin, Madison, Wisconsin, United States of America; 5 Laboratory of Optical and Computational Instrumentation, University of Wisconsin, Madison, Wisconsin, United States of America; Fox Chase Cancer Center, United States of America

## Abstract

The utilization of 3D, physiologically relevant in vitro cancer models to investigate complex interactions between tumor and stroma has been increasing. Prior work has generally focused on the cancer cells and, the role of fibroblast culture conditions on tumor-stromal cell interactions is still largely unknown. Here, we focus on the stroma by comparing functional behaviors of human mammary fibroblasts (HMFs) cultured in 2D and 3D and their effects on the invasive progression of breast cancer cells (MCF10DCIS.com). We identified increased levels of several paracrine factors from HMFs cultured in 3D conditions that drive the invasive transition. Using a microscale co-culture model with improved compartmentalization and sensitivity, we demonstrated that HMFs cultured in 3D intensify the promotion of the invasive progression through the HGF/c-Met interaction. This study highlights the importance of the 3D stromal microenvironment in the development of multiple cell type in vitro cancer models.

## Introduction

Cancer cells cultured in an extra cellular matrix (ECM) (often called three-dimensional (3D) culture) show differences in functional behaviors such as differentiation, proliferation, and gene expression [1-3], when compared to cells cultured on a flat surface (two-dimensional (2D)). The growing consensus is that 3D models recreate key aspects of the microenvironment more faithfully and, in some cases, provide more comprehensive and relevant biological information that is impossible or difficult to obtain from 2D models [4-6]. This realization has prompted increased use and exploitation of 3D culture for in vitro cancer models [3,7-9]. One hypothesis attributes the changes observed in 3D culture to the enhanced interactions between cells and the surrounding ECM. This hypothesis is supported by reports of a growing number of different signaling mechanisms in 3D microenvironments compared to 2D microenvironments over the last decade [7,9-12]. However, there are still relatively few studies directly comparing 2D vs. 3D in vitro systems. In addition, while the role of the matrix in regulating fibroblast behavior has been previously studied, the consequences of modified fibroblast behavior via paracrine signaling with cancer cells is less well understood. Co-culture of cancerous cells with stromal fibroblasts has been shown to induce significant changes in tumor development and progression. Fibroblasts surrounding a pre-invasive tumor can become activated and play a critical role in the progression to invasion via enhanced secretion of cytokines, growth factors, and proteases such as TGFβ1, HGF, SDF-1, and MMP2 [13-15]. Particularly in breast cancer, the progression from ductal carcinoma in situ (DCIS) to invasive ductal carcinoma (IDC) is believed to be actively driven by complex interactions with the surrounding microenvironment including interactions with various stromal fibroblasts [16-20]. In this study, we focus on examining the paracrine interaction between cancer cells and stromal fibroblasts during the breast cancer progression from DCIS to IDC in the context of matrix effects on the stromal cells and their subsequent regulation of cancer progression.

To obtain a more comprehensive understanding of the complex tumor-stroma interactions during breast cancer progression, it is critical to develop a more holistic view of the effect of the microenvironment on the interaction between multiple cell types. Current studies, based on platforms such as the transwell or multiwell assay, focus primarily on the tumor cell, while neglecting to consider the culture environment of the co-cultured fibroblast cells. Further, these models have limited functionality when investigating more complex mechanisms including paracrine/autocrine signaling, cell-cell physical interactions, and matrix-cell interactions. Microfluidic models have been shown to provide a higher level of control over the microenvironment, noticeably through the ability to control ECM and soluble-factor signaling cues separately [21-26]. For example, we recently developed an in vitro co-culture model of stromal and cancer cells that supports the progression from DCIS to IDC using a simple microfluidic system [27]. Importantly, the microfluidic system is capable of mimicking the microenvironment more precisely than conventional systems enabling lines of inquiry that are difficult to pursue using traditional systems. To date, however, the conditions of stromal fibroblast culture are rarely considered in these models, and, to the best of our knowledge, have not been mechanistically well assessed.

In this study, we examined the influence of 2D and 3D culture of human mammary fibroblasts (HMFs) on the invasive transition of breast cancer cells (MCF10-DCIS.com (MCF-DCIS) cells), specifically known as the DCIS to IDC transition. We show that when HMFs are cultured in a 3D matrix, they secrete more paracrine signaling molecules than in 2D culture conditions and that these molecules increase the invasive behavior in DCIS cells. First, we collected conditioned media from 2D and 3D cultures of HMFs and measured the degree of invasive transition of MCF-DCIS cells in the different conditioned media. Second, we analyzed the mRNA expression of five stromal fibroblast-derived molecules (CXCL12, MMP14, HGF, COX2, and TGFβ1) of HMFs cultured in 2D and 3D conditions. Bead-based ELISA was performed to profile the concentrations of eight secreted proteins in 2D and 3D conditions. Among the examined molecules, HGF was selected for further investigation because of its known effect in the invasion of cancer cells, particularly through its ability to activate c-Met. HGF/c-Met signaling was further validated by adding a neutralizing antibody against HGF and a small molecule inhibitor that inhibits c-Met phosphorylation. Finally, we developed and applied a 3D microfluidic platform to perform 3D and 2D combined co-culture of MCF-DCIS cells and HMFs to validate the data obtained in the conditioned medium experiments using a more holistic model. This work underscores the importance of a 3D microenvironment in paracrine interactions, identifies important factors that influence progression and whose expression is increased in 3D culture and validates micro culture models as a useful tool enabling advanced studies.

## Results and Discussion

**Figure 1:**
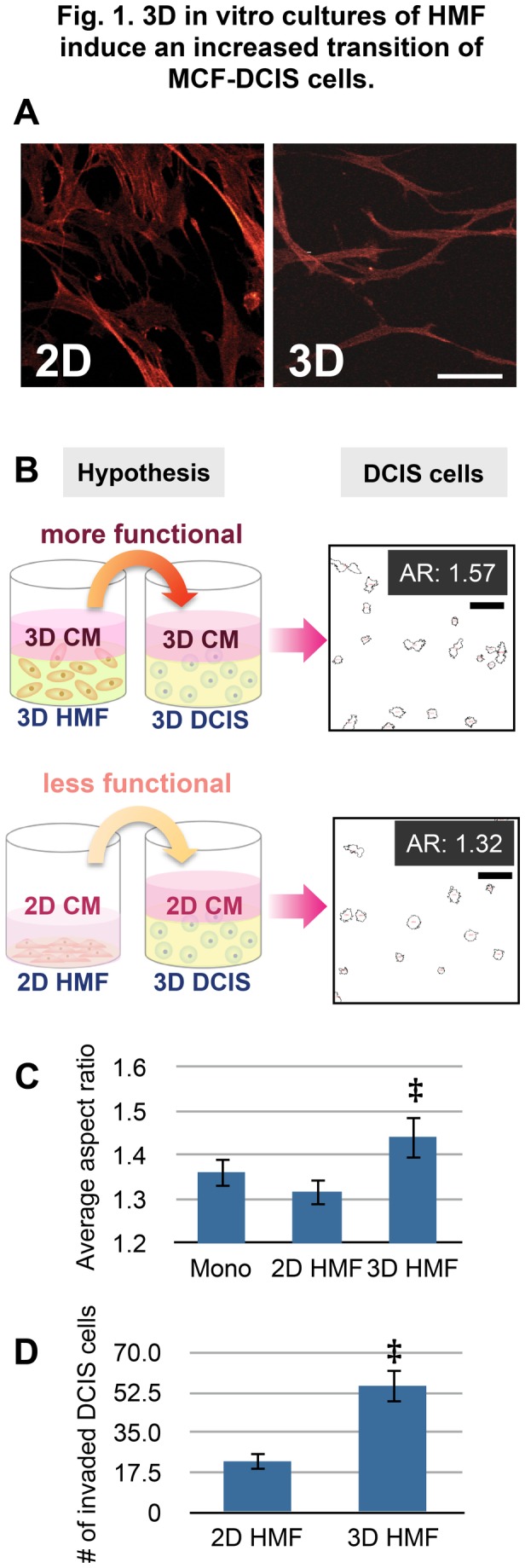
3D in vitro culture of HMF induces an increased transition of MCF-DCIS cells. (A) Different morphologies of HMFs in 2D vs. 3D conditions. These images clearly show that HMFs in 3D have more fiber-like structures. The scale bar represents 60 µm. (B) Conceptual illustration of the difference of HMF behaviors in 2D and 3D. The conditioned medium collected from 3D culture of HMF (3D CM) stimulates invasive transition more than the conditioned medium collected from 2D culture of HMF (2D CM), and stimulates more invasive transition of MCF-DCIS cells in 3D. Outlines of MCF-DCIS clusters cultured in 3D mixed matrix with 3D CM and 2D CM. The clusters cultured with 3D CM produced more elongated clusters with aspect ratio (AR) 1.57. Scale bar is 100 μm. (C) Bar graph showing average aspect ratio of MCF-DCIS clusters cultured with control (serum free medium, mono), 2D HMF (co-cultured with HMFs in 2D), and 3D HMF (co-cultured with HMFs in 3D). ‡ represents p value of 0.048. (D) Bar graph showing data obtained from transwell invasion assays with conditioned media from 2D culture of HMF (2D HMF) and 3D culture of HMF (3D HMF). ‡ represents p value of 0.022.

MCF-DCIS cells show the ability to replicate key aspects of breast cancer progression from DCIS to IDC [16,28,29]. This transition has further been shown to be facilitated by co-culture with fibroblasts, particularly when fibroblasts are cultured in 3D conditions versus 2D conditions [27]. As shown in Figure 1A, HMFs show clearly very different morphologies depending on whether they were in 2D or 3D conditions; HMFs in a 3D condition show thinner and more fiber-like morphology (as shown in Fig. 1A/3D), while HMFs in a 2D condition show more widely spread morphology (as shown in Fig. 1A/2D). As reported by Grinnell, it is possible that the stiffness of collagen matrices could be responsible for the different functional behavior of fibroblasts in 3D compared to the fibroblasts in 2D conditions [12,30]. However, the different functional behaviors of fibroblasts in 2D vs. 3D conditions are not clearly known. Here, we propose a mechanistic assessment of the effects of 2D and 3D culture conditions on the functional activity of HMFs and their subsequent impact on the invasive transition of MCF-DCIS cells using both established macroscale methods and emerging microscale methods.

### HMFs cultured in 3D induce a more invasive transition of MCF-DCIS cells than HMFs cultured in 2D conditions

We first assessed functional differences of HMFs cultured in 2D and 3D conditions by comparing the amount of secreted signaling molecules from HMFs present in culture media. Further, the effect of HMFs cultured in 2D and 3D conditions on the invasive transition of MCF-DCIS cells was investigated. To examine effects solely caused by soluble molecules in each condition, conditioned media from 2D and 3D cultures of HMFs was collected after 48 hours of culture in 48 well-plates and added to 3D cultures of MCF-DCIS cells in 48 well-plates (Figure 1B). For 3D culture, we used the mixture of collagen I and Matrigel because we had previously found that this mixed matrix condition was suitable to culture both MCF-DCIS cells and HMFs and to induce invasive transition of MCF-DCIS cells [27]. The transition of MCIS-DCIS cells to an invasive phenotype was evaluated using two well-established measures: the aspect ratio (AR, major axis over minor axis of cancer cell clusters) and the degree of invasion in transwells. The aspect ratio is one of the established measures for estimating the degree of invasive transition of cancer cells [27]. Conditioned media from HMF cultured in 3D induced a more invasive transition of MCF-DCIS cells (Figure 1C), which displayed more elongated clusters (i.e., higher aspect ratio). Additionally, transwell invasion assays showed a higher invasion of MCF-DCIS cells when stimulated by 3D conditioned media than by 2D conditioned media (p=0.022) (Figure 1D). These observations suggest that increased secretions of specific signaling molecules or decreased secretion of inhibitory molecules from fibroblasts occur in 3D conditions, and these stimulate the invasive transition of MCF-DCIS cells.

**Figure 2:**
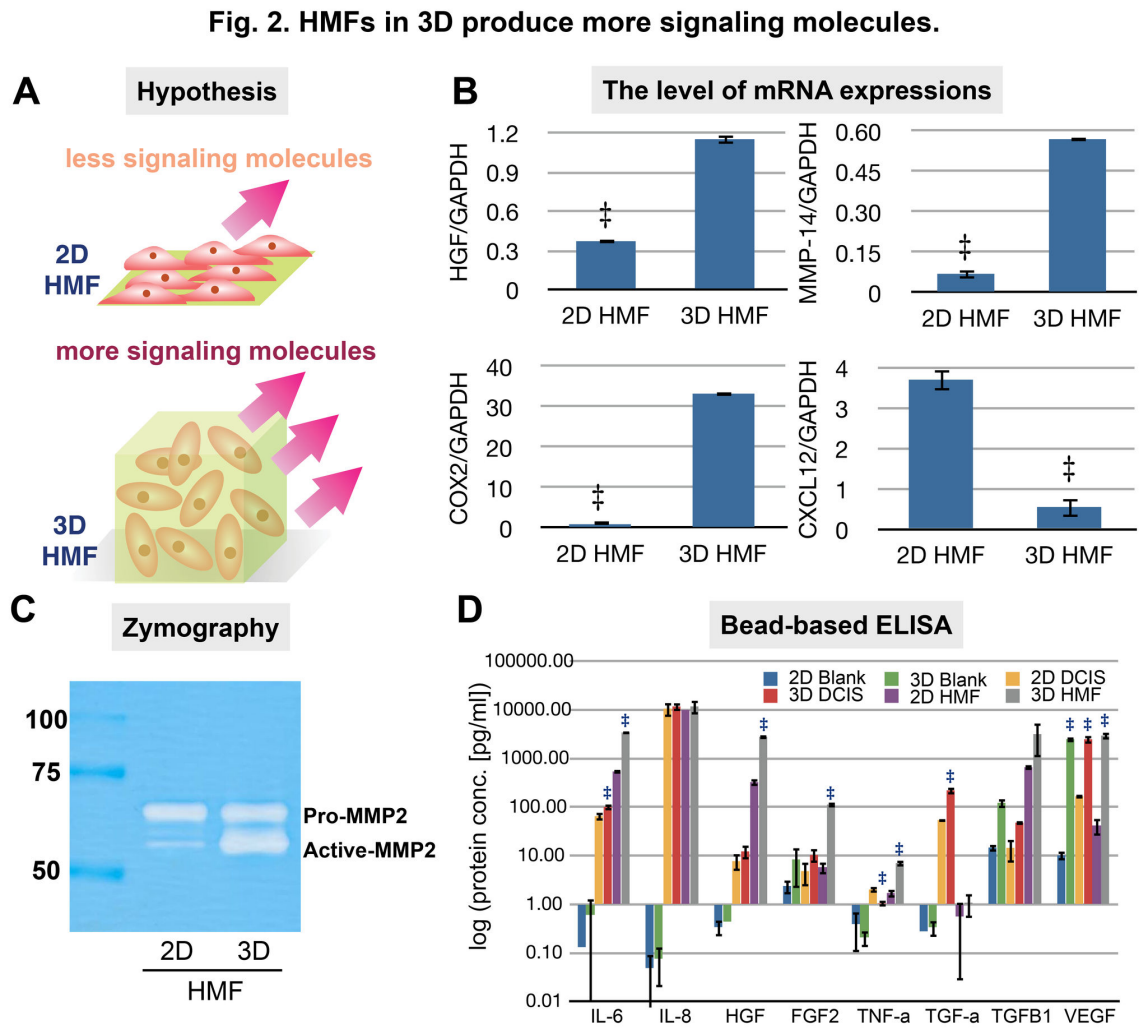
HMFs in 3D produce more signaling molecules. (A) Conceptual illustration showing HMFs in 3D produce more signaling molecules. (B) Bar graphs showing the mRNA expressions of HGF, MMP14, COX2, and CXCL12 in HMFs cultured in 2D and 3D conditions. ‡ represents a p value of less than 0.05. (C) Zymography showing the presence of increased active MMP2 in the 3D conditioned medium of HMFs. (D) Bead-based ELISA showing the concentrations of target proteins in conditioned media collected from 3D and 2D cultures of HMFs and MCF-DCIS cells.

To identify which molecules are secreted at different levels in 3D conditions (Fig. 2A), we analyzed 1) mRNA levels, 2) gelatinase (MMP2) activity, and 3) concentrations of secreted proteins from HMFs cultured in 2D vs. 3D. First, we selected five stromal derived molecules (HGF, COX2, MMP14, TGFβ1, and CXCL12) based on previously published studies [13,17,31-36]. The mRNA expressions of the selected molecules in HMFs cultured in 2D and 3D conditions were quantified after 48 hours cultivation in each condition. Because HMFs proliferate faster under 2D conditions, 2D samples were loaded at a lower density in order to achieve similar final cell densities as compared to the 3D samples at the collection time (48 hours) (Fig. S1). This proliferation difference is consistent with a previous study led by Su et al. [37]. We measured the integrated fluorescent intensity after nuclear staining to estimate the total number of cells in each condition. Our calibration curves shown in Figure S1C verified that the integrated intensity was linearly proportional to the total number of cells (R^2^ values are 0.9895 for 2D conditions and 0.9972 for 3D conditions). In addition, to achieve the same cell number to media volume ratio, we added the same volume of a serum-free medium to each condition. Among the five molecules tested, HGF, MMP14, and COX2 showed higher expression from HMFs cultured in 3D conditions compared to HMFs cultured in 2D conditions. CXCL12 showed an opposite trend (Fig. 2B). TGFβ1 expression levels were not significantly different between HMFs cultured in 2D and 3D conditions. Second, using zymography, we found that active MMP2 secretion was higher in HMFs cultured in 3D conditions (Figure 2C). We examined the effect of different gel densities on 3D conditions by testing a range of gel densities and proliferation effects (since 2D conditions induce increased cell growth) by testing different cell seeding densities. All of these conditions displayed similar trends (Figure S1). As no significant differences were observed for the different cell and collagen densities tested, we chose a high cell density for the 2D conditions (6x10^4^cells/well) and a lower concentration of the 3D mixed matrix (50:50 Matrigel: collagen I, the final concentration of collagen I -0.8mg/ml) for all subsequent experiments.

Finally, bead-based ELISA was used to quantify the concentrations of eight secreted proteins (HGF, IL6, IL8, FGF2, TNFα, TGFα, TGFβ1, VEGF) from HMFs as well as from MCF-DCIS cells in 2D compared to 3D. The results showed that seven molecules (out of eight) were secreted in higher concentrations from HMFs in 3D than in 2D (Figure 2D). MCF-DCIS cells, on the other hand, secreted relatively similar amounts of the eight proteins analyzed whether cultured in 2D or 3D. Interestingly, this suggests that, with regard to the secreted factors examined, HMFs are more affected by culture conditions than DCIS cells. In addition, blank hydrogel controls (mixture of Matrigel and collagen) show a significant amount of IL6, IL8, TGFβ1, and VEGF without cells. These data verify the fact that Matrigel contains various growth factors, and it is possible that these growth factors might also activate HMFs in 3D conditions. Therefore, for the following experiments we used proper controls such as collagen I only controls in order to determine whether there was any influence from the Matrigel.

These observations support our hypothesis that 3D in vitro culture of HMF activates secretion of soluble paracrine signaling molecules that influence the invasive transition of MCF-DCIS cells. We further explored the influence of hepatocyte growth factor (HGF) on DCIS progression to IDC because HGF is a well-known scattering factor, a major contributor for invasive growth of cancer cells [38-40]. Jedeszko et al., for example, showed that, using a conventional 3D in vitro model and an in vivo model, mammary fibroblasts engineered for amplified HGF-secretion increased the percentage of DCIS structures with invasive outgrowth and activated c-Met [38]. However, their work did not compare the effect of fibroblasts cultured in 3D conditions and in 2D conditions on the scattering effect of DCIS in 3D in vitro systems.

### Fibroblast-derived HGF production is increased in 3D in vitro culture and is necessary for progression of MCF-DCIS cells from a non-invasive to invasive phenotype.

HGF is a multi-functional cytokine stimulating invasion, motility, morphogenesis, as well as metastasis and is known to act through its specific receptor, c-Met on cancer cells [41-46]. Further, over-expression of HGF has been detected in various invasive carcinomas, including breast carcinomas, and high expression of HGF has been identified as a predictor of recurrence and shortened survival in breast cancer patients [39].

**Figure 3:**
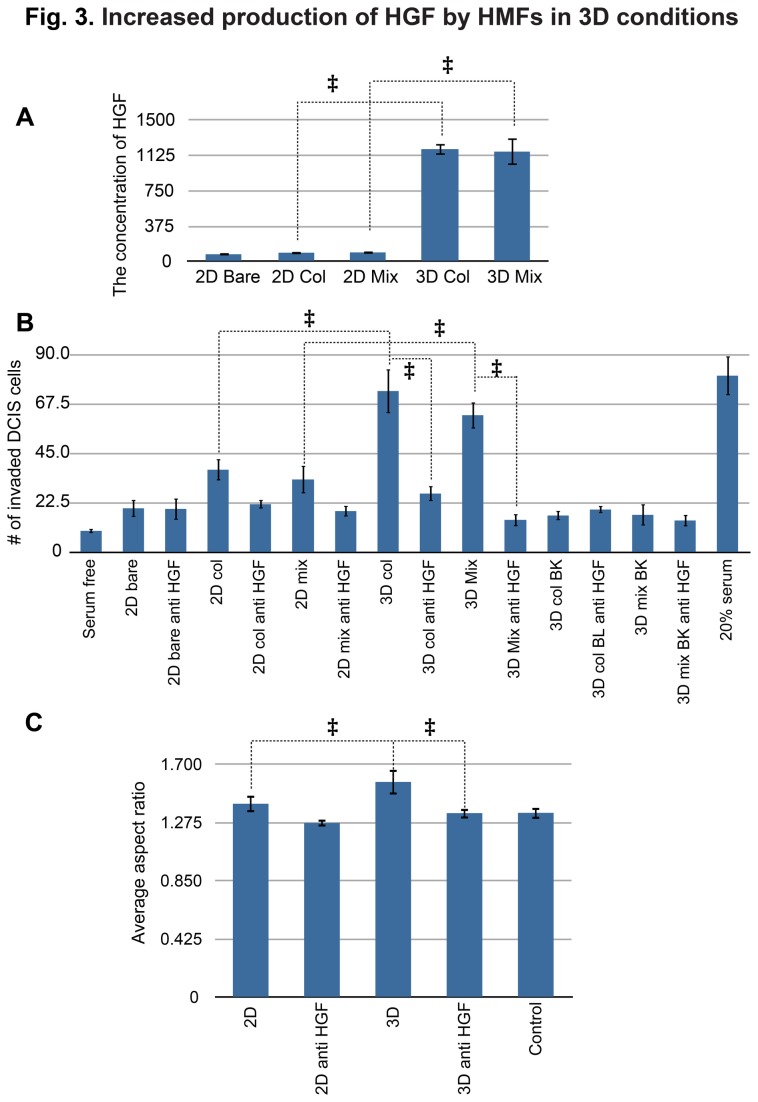
Increased production of HGF by HMFs in 3D conditions. (A) An ELISA assay showing the concentration of HGF [pg/ml] in different conditioned media collected from three 2D conditions (bare, collagen-coated, mixed matrix-coated), and two 3D conditions (collagen I only, mixed matrix). HMF cells in all 3D conditions significantly increased the secretion of HGF. ‡ represents a p value of less than 0.05. (B) Invasion of MCF-DCIS cells with HGF neutralizing antibody (anti HGF) using transwells. The HGF neutralizing antibody (0.5 μg/ml) is added to various 2D CM, 3D CM and BK CM (the conditioned medium collected from blank mixed gels). A serum free medium is used as a negative control and a 20% serum-containing medium is used as a positive control. ‡ represents p value of less than 0.05. (C) The aspect ratio of MCF-DCIS cells cultured in a 3D condition with 2D CM and 3D CM with or without the HGF neutralizing antibody. ‡ represents a p value of less than 0.05.

In our co-culture system, HMFs were the main source of HGF. We measured HGF mRNA expression in MCF-DCIS cells in both 2D and 3D conditions and found that it was not detectable under any conditions (Figure S2). Further, blank matrix did not release significant amounts of HGF (Figure 2D). Thus, we concluded that the main source of HGF originates from the HMF cells. In order to compare HGF production by HMF cultured in 2D vs. 3D conditions, we conducted ELISA assays using various conditioned media. We collected conditioned media from three different 2D conditions (i.e., bare surface, collagen-coated surface, mixed matrix-coated surface) in order to examine whether the presence of matrix proteins could increase the production of HGF by HMF cells. In addition, 3D conditioned media from the collagen I only matrix was examined in order to verify whether there was any influence from the Matrigel. As shown in Figure 3A, HMFs in 3D conditions consistently produced more HGF than in 2D conditions. We also found that the production of HGF was constant over the culture period, as the concentration of HGF at 48 hours was roughly double that of 24 hours (data not shown).

Next, we examined the effect of HGF on MCF-DCIS transition by inhibiting HGF activity with an HGF neutralizing antibody. The effect of HGF inhibition was estimated by conducting invasion assays and morphology analyses. The same conditions applied to experiments shown in Figure 1 were added to the various experimental conditions shown in Figure 3 in order to efficiently present the effect of HGF inhibition. 50 μg/ml of HGF neutralizing antibody was added to the conditioned media collected from various 2D and 3D HMF cultures. The addition of neutralizing antibody to the 3D conditioned media reduced the number of invaded cells to the level of the negative control (Figure 3B). The conditioned media that did not contain the HGF neutralizing antibody displayed significantly higher invasion. As shown in Figure 3B, we found that the presence of matrix proteins (both collagen and Matrigel) in 2D cultures slightly increased the number of invaded DCIS cells, but we also noticed that the differences were not statistically significant (P>0.05). The DCIS cells showed less invasion with the HGF neutralizing antibody added to the 2D conditioned media; however the reduction of invasion was not statistically significant (P>0.05). The morphologies of MCF-DCIS cells in the 3D conditioned medium showed a similar trend. The MCF-DCIS cells cultured in the conditioned medium collected from a 3D culture of HMFs showed a more elongated morphology (i.e., a higher aspect ratio) compared to the cells cultured in the conditioned medium collected from a 2D culture of HMFs (Figure 3C). The addition of HGF neutralizing antibodies into the 3D conditioned medium reduced the aspect ratio considerably. These results indicate that HGF is a main paracrine factor secreted from HMFs modulating the invasion of MCF-DCIS cells and is up-regulated in 3D conditions, and that removal of this factor rescues the non-invasive phenotype.

### Microfluidic 3D co-culture platform recapitulates the 2D/3D fibroblast effect observed in macroscale, allowing additional functional endpoints and enabling improved parametric control

To further validate the difference between HMFs cultured in 2D and 3D, we designed a microfluidic 3D co-culture platform that allows one to mix and match 2D and 3D co-cultures with short diffusion distances between the cell types. The system allowed us to co-culture MCF-DCIS cells in 3D with HMF cells in either 2D or 3D. Transwell systems have traditionally been used to perform combined 2D and 3D co-culture. However, these systems are limited in their ability to monitor the changes in both cell types in a single experiment, require relatively large numbers of cells, and significant quantities of expensive matrix proteins (e.g. collagen, Matrigel).

Microfluidic co-culture platforms provide additional capabilities over conventional transwell systems. The microscale systems allow a reduction of about 100 fold in cells and reagents use, saving resources, enabling an increase in the number of endpoints, and enabling higher sensitivity to paracrine factors [47]. The major difference between macro and micro systems is summarized in Table S1. Additionally, the ability of microsystems to horizontally compartmentalize allows the monitoring of changes in cells and their associated ECM [27]. Second harmonic generation (SHG) is a powerful imaging technique that is becoming widely used to conduct label-free imaging of collagen and capture intrinsic characteristics of collagen networks [48-51]. In our study, we used SHG intensity to further define the invasive phenotype of the MCF-DCIS clusters (i.e., more invasive MCF-DCIS clusters alter ECM architecture at higher degree and exhibit higher SHG intensity values) [27].

**Figure 4:**
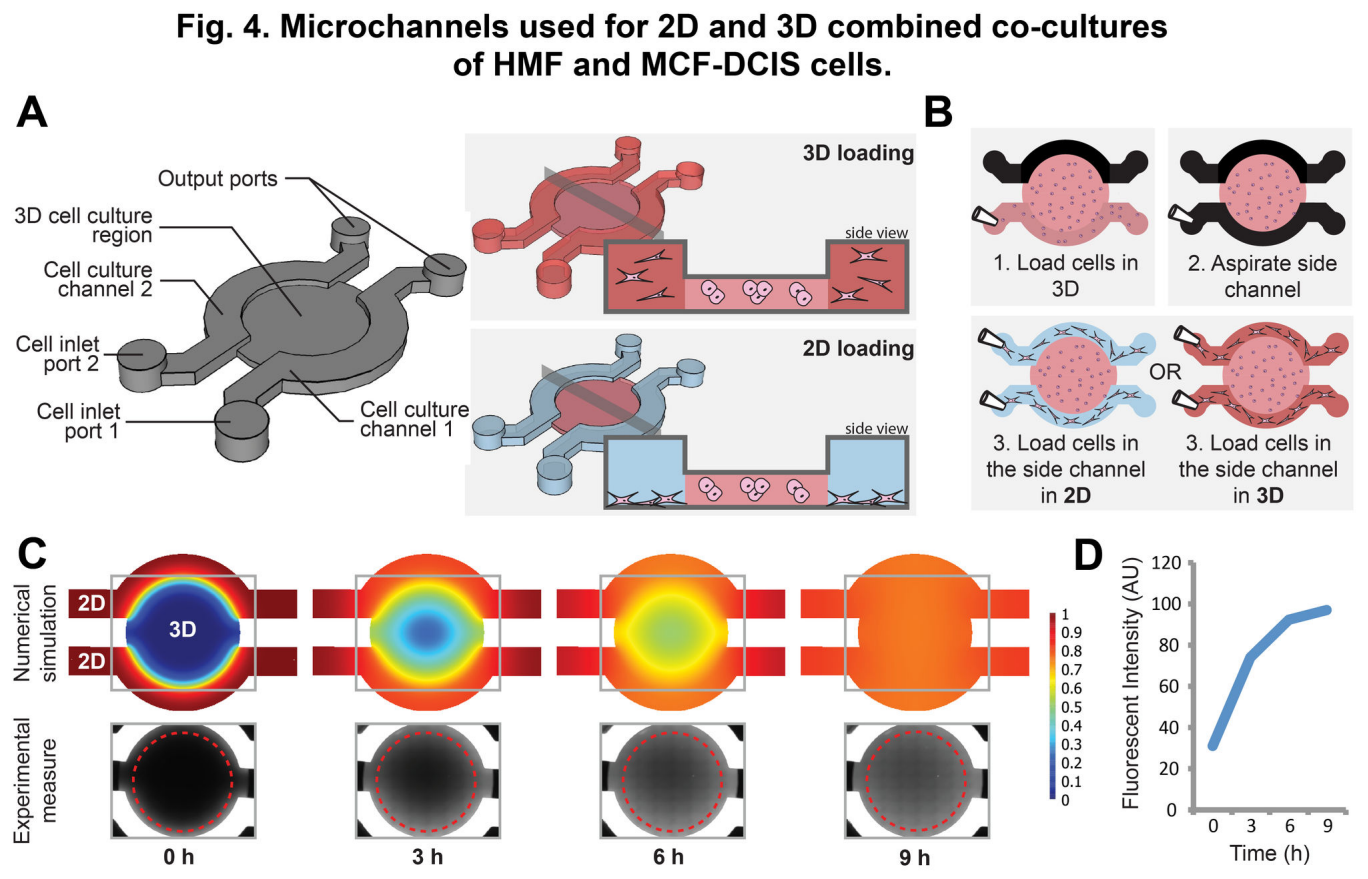
Microchannels used for 2D and 3D combined co-cultures of HMF and MCF-DCIS cells. (A) 3D schematic and cross-section of the microchannels used for 2D and 3D combined co-culture of HMF and MCF-DCIS cells. (B) Illustrations of the loading process showing the simplicity of loading both in 2D and 3D conditions. (C) Visualization of the diffusion process in the microdevice using a numerical COMSOL simulation and a timelapse microscopy of AlexaFluor488-Dextran10kD dye. (D) Average fluorophore concentration in the inner chamber of the microdevice plotted through time.

We designed a simple compartmentalized microfluidic system composed of three connected cell-culture chambers: a central chamber for 3D culture of MCF-DCIS cells, and two outer chambers for 2D or 3D culture of HMFs (Figure 4A). The central chamber was designed with a lowered height to facilitate pinning of fluid in that region [52], such that the fluid can be flowed into the central chamber from either side chamber and be passively retained when fluid is aspirated from either side chamber (Figure 4A,B, Figure S3, Figure S4, and movie S1). The surface areas of the center chamber and the two side chambers were designed to be roughly identical (table S1). The sample loading was completed in 3 simple steps (i.e., first injection, aspiration, and second injection), and did not require the use of fluids with matching viscosities as other laminar flow patterning based devices do [27]. The tubeless microfluidic method utilized for driving fluid flow is readily compatible with common pipetting methods, allowing increased throughput assays using a small number of cells [53-57]. To show that the signaling molecules can diffuse well from the side chamber to the center chamber within the time frame of the experiment (i.e., six days), we characterized the diffusion timescale and pattern of the device by conducing a fluorescent dye loading experiment using the fluorophore Texas Red bound to Dextran 70K MW, the approximate size of the HGF (Figure 4C and 4D, Figure S5). The outcomes of the simulation show that the produced paracrine factors in one compartment diffuse to the other compartment and ensure paracrine interaction during the six-day culture period.

**Figure 5:**
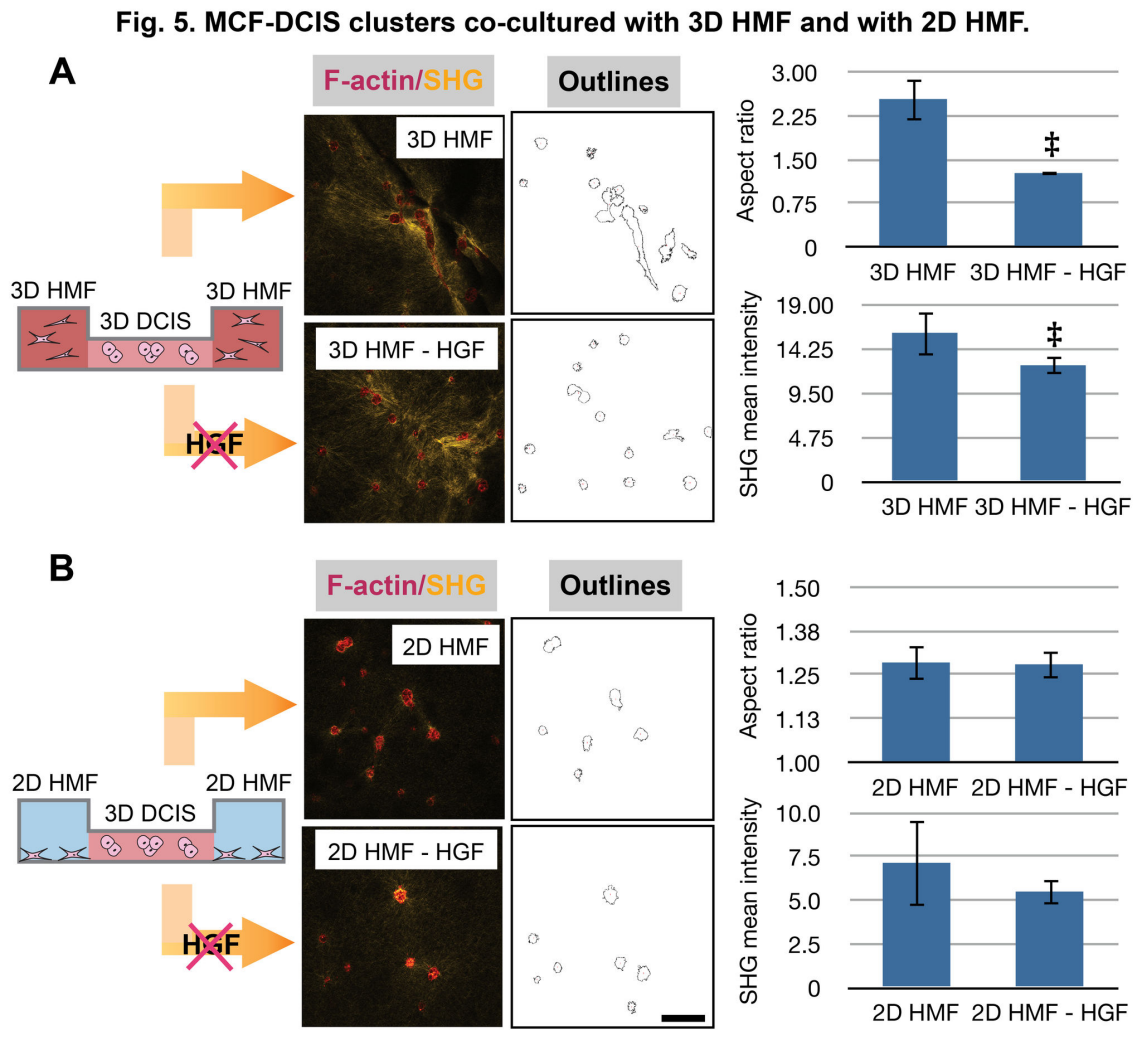
MCF-DCIS clusters co-cultured with 3D HMF and with 2D HMF. (A) MCF-DCIS clusters (red and outlines) co-cultured with 3D HMF and neutralizing HGF antibody at 0.5 μg/ml (3D HMF-HGF). SHG (yellow) shows changes in collagen architecture around MCF-DCIS cells. The addition of HGF neutralizing antibody significantly decreased the aspect ratio of MCF-DCIS cells and the mean intensity of SHG. ‡ represents p value less than 0.05. (B) MCF-DCIS clusters (red and outlines) co-cultured with 2D HMF and neutralizing HGF antibody at 0.5 μg/ml (2D HMF-HGF). Scale bar is 100 μm.

The interaction of HGF and c-Met receptor was investigated by adding both HGF neutralizing antibody and c-Met inhibitor to examine whether blocking of either HGF or c-Met reduces the invasive transition of MCF-DCIS cells [44,58]. After 6 days of cultivation, samples were fixed and the morphology of MCF-DCIS clusters as well as SHG intensity were analyzed. The addition of the HGF neutralizing antibody or c-Met inhibitor to the 3D HMF/3D MCF-DCIS co-culture significantly decreased the invasive transition of MCF-DCIS cells as quantified by the decreased AR of the clusters (Figure 5A, Figure S6A). Interestingly, while the addition of the HGF neutralizing antibody significantly reduced the mean SHG intensity around MCF-DCIS cells, the inhibition of c-Met on MCF-DCIS cells did not significantly alter the mean intensity of SHG (Figure 5A, Figure S6A). Based on the AR data shown in Figure 3C and Figure 5A, we observed that MCF-DCIS cells in co-culture with 3D fibroblasts presented a higher AR value than MCF-DCIS cells cultured in the conditioned medium collected from 3D fibroblasts (approximate AR values of 2.5 vs 1.5, p<0.05). Additional ECM remodeling caused by nearby fibroblasts in co-culture may be responsible for the increased AR change in co-culture. Moreover, secreted signaling proteins in co-culture might be more concentrated and activated than the protein preserved in conditioned media, thus inducing more invasive transition of MCF-DCIS cells. This difference may support a hypothesis that the invasive transition of MCF-DCIS cells are not solely governed by soluble factor interaction but also regulated by mechanical interaction in 3D conditions. The changes in morphology and SHG intensity were negligible when the antibody and inhibitor were added to the 2D HMF/3D MCF-DCIS co-culture (Figure 5B, Figure S6B). This result is consistent with the previous findings that HMFs in 2D produce significantly lower amounts of HGF and correspondingly induce less activation of the c-Met pathway. In addition, we did not find a link between integrin β1 function and HGF production in this system in experiments utilizing integrin β1 blocking antibodies (Figure S7), suggesting that β1 integrin itself may not strongly contribute to the production of HGF. Based on the fact that there are 17 α subunits and 8 β subunits of integrins and these α and β subunits heterodimerize to produce 22 different complexes [59,62], it was not surprising to find that blocking one specific integrin did not disturb complex interactions between HMFs and various ECM compositions in the mixed matrix used in this work. Alternatively, integrins may play no role in regulating the secretion of HGF. Together, these findings show that stromal fibroblasts do participate in the invasive transition of tumor in vitro, but also that their culture conditions and microenvironmental cues are paramount in enabling that effect. Importantly, the increased throughput, smaller volumes and lower reagent costs associated with the microscale culture platform will facilitate further “screening” investigations with integrins and other potential players to speed our understanding of the complex mechanisms involved in these phenomena.

## Conclusions

The transition from DCIS to IDC is a critical stage in breast cancer progression, and improved understanding of the signaling mechanisms that regulate this transition can have clinical impact by identifying potential targets for alternative treatment options. The development and validation of models to study the invasive transition of breast cancer is central to advancing our understanding of the fundamental mechanisms involved. While the importance of 3D culture in in vitro systems and the influence of stromal fibroblasts in DCIS progression have been previously reported, this work provides strong evidence that the 3D environment itself affected stromal fibroblasts. The 3D culture of fibroblasts results in an increased secretion of signaling molecules compared to stromal fibroblasts cultured in 2D, subsequently enhancing the progression towards invasive phenotypes of the breast cancer cells. We have identified functional differences in HMF cultured in 2D vs 3D conditions. Specifically, the expression of HGF by HMF cultured in 3D is increased resulting in the transition of DCIS to IDC.

Further, we developed a microfluidic in vitro system to provide a more efficient and physiologically relevant platform for the investigation of complex mechanisms involved in the cell-3D environment interaction. The microfluidic system enabled combined 2D/3D co-culture of MCF-DCIS and HMF cells using a simple pipette-driven loading process. Moreover, the side-by-side co-culture improved imaging capabilities by minimizing interference from the other cell type. The small volume required per endpoint and the compatibility with existing high-throughput infrastructure enables the use of various neutralizing antibodies and small molecule inhibitors with minimal cost and labor enabling screening approaches in 3D culture.

## Materials and Methods

### Cell culture

Human mammary fibroblast (HMF; originally termed RMF/EG) cells were provided by Dr. Kuperwasser [60] and were cultured in DMEM with high glucose and L-glutamine (Invitrogen, 11965-092, Grand Island, NY) supplemented with 10% calf serum (Invitrogen, 26010074, Grand Island, NY), and penicillin/streptomycin. MCF10-DCIS.com cells [31] were purchased from Asterand (Detroit, MI), and were cultured in DMEM-F12 with L-glutamine (Invitrogen, 11965-092, Grand Island, NY) supplemented with 5% horse serum (Invitrogen, 11320-033, Grand Island, NY), and penicillin/streptomycin. For the co-culture experiments, we used the same medium that was used to culture MCF-DCIS cells. Previously, we tested culture media for co-culture conditions and found that fibroblasts were not very sensitive to media conditions [27]. All cultures were maintained at 37 °C in a humidified atmosphere containing 5% CO2.

### Cell line authentication

MCF10DCIS.com cells were authenticated by using “Cell Check” service provided by RADIL(http://www.radil.missouri.edu) on the date of September 26, 2011. The sample was confirmed to be of human origin and no mammalian inter-species contamination was detected. The alleles for 9 different markers were determined and the results were compared to the alleles reported for a previously submitted sample from Asterand. The genetic profile for the our sample was identical to the genetic profile of the Asterand sample reported previously.

### Microchannel design, fabrication, and operation

The microfluidic devices were fabricated using multilayered SU-8 molds and PDMS-based soft-lithography. In brief, three layers of SU8-100 (Microchem Corp), of thicknesses 100 µm, 150 µm, and 500 µm, were spun on a 150 mm diameter silicon wafer and patterned according to the manufacturer’s guidelines. UV lithography was performed using an Omnicure 1000 light source (EXFO) using masks printed on transparency (ImageSetter, Madison, USA). Subsequently, the wafer was developed using SU-8 developer (PGMEA, Sigma) and cleaned in acetone and IPA. Polydimethylsiloxane (Sylgard 184, Dow Corning) was mixed in a 1:10 cross-linker to base ratio, degassed for 30 min, and poured over the clean wafer on a hot plate. The molding process was performed by layering a transparency film, a layer of silicone foam, a 75 mm by 100 mm slab of glass, and a 5 kg weight on top of the wafer and PDMS, and baking the stack at 80°C for 3 hours. The cured PDMS layers were peeled off of the wafer, sterilized in 70% ethanol, and attached to polystyrene cell culture dishes (TPP AG, Switzerland). For multiphoton and confocal laser scanning microscopy, PDMS channels were attached to a glass bottom culture dish (P50G-0-30-F, MatTek corp, Ashland, MA) after treating both the PDMS layer and the petri dish in a plasma chamber for 50 seconds at 100W.

The channels were placed on ice for the loading and a cell suspension containing MCF-DCIS cells and a mixed matrix was loaded into one of the input ports until the fluid filled the center circular chamber. The excess cells in the side channels were removed by applying a gentle vacuum to the loading port. The cells-in-gel suspension was polymerized in a cell-culture incubator for 10 min by manually flipping the channels upside down every 2 min to prevent cell settling. The two side chambers were loaded with either a cell suspension of HMF cells in a medium, in a mixed matrix, or with blank gel.

### Surface coating for in vitro 2D cultures

For a collagen I-coated surface, an acidic collagen I solution (Collagen I, High concentration, rat tail, 354249, BD Biosciences) was diluted in 1x PBS at a concentration of 100 µg/ml. For a mixed matrix-coated surface, the growth factor reduced Matrigel (Basement Membrane Matrix, Growth Factor Reduced (GFR), Phenol-Red-free, 10ml*LDEV-free, 356231, BD Biosciences) was added to the diluted collagen I solution (100 µg/ml) at the dilution ratio of 1:10. Prepared solutions were added to cell culture dishes and incubated for 1 hour at room temperature. After incubation, the remaining solutions were removed and rinsed three times with 1x PBS prior to loading cells.

### Sample preparation for in vitro 3D culture

Collagen was prepared initially at a concentration of 5.0 mg/ml by neutralizing an acidic collagen solution (Collagen I, High concentration, rat tail, 354249, BD Biosciences) with 100mM HEPES buffer in 2X PBS (pH 7.7). For the collagen I only matrix condition, cells and a culture medium were added to neutralized collagen I gel to achieve a final concentration of 1.6 mg/ml. For mixed gel conditions, neutralized collagen gel and Matrigel (Basement Membrane Matrix, Growth Factor Reduced (GFR), Phenol Red-free, 10 ml *LDEV-Free, 356231, BD Biosciences) were mixed in equal volumes, and the collagen I concentration (0.8 mg/ml and 2.0 mg/ml) was adjusted by cell suspension and a culture medium. For loading into microfluidic channels, the neutralized sample was kept at 4 °C for at least 15 min to apply an additional time for nucleation before channel loading [61].

### Conditioned media collection

HMFs cultured in 2D proliferate faster than HMFs cultured in 3D, and, accordingly, we prepared lower cell densities for 2D samples (3x10^4^ cells/48-well and 6x10^4^ cells/48-well) than the density of 3D samples (1.2x10^5^ cells/48-well) in order to obtain similar final cell densities in the 2D and 3D samples after 48 hours. After cells were completely adhered to culture plates (for 2D samples) and to ECM (for 3D samples), the 400 µl of a serum-free DMEM medium were added on top of samples. After 24 and 48 hours, conditioned media were collected and were centrifuged at 4000rpm for 5 min to pellet any floating cells and debris.

### Invasion assay

The invasiveness of MCF-DCIS cells was assayed by using transwell invasion chambers (Matrigel Invasion Chambers in two 24-well plates, 8.0 µm, 354480, BD Biosciences). We resuspended MCF-DCIS cells in serum-free DMEM/F12 (5x10^4^cells/ml), and seeded in the upper compartment of the chamber (0.2ml per chamber). The lower compartment was filled with 0.75ml of DMEM/F12 supplemented with different conditioned media collected from 2D and 3D cultures of HMF as a chemoattractant. After incubation at 37°C in a humid atmosphere for 36 hours, filters were rinsed with PBS. Remaining cells on the upper surface were wiped away with a wet cotton swab, and those on the lower surface were fixed with 4% paraformaldehyde, and stained with Hoechst (Hoechst 33342, H3570, Molecular Probes). The number of invaded cells per microscopic view was counted and averaged.

### Proliferation assays

For proliferation assays, 2D and 3D samples were fixed at each time point (0, 24 hours, and 48 hours) and nuclei stained with ToPro3. Cells were washed with 1xPBS then fixed with 4% paraformaldehyde for 30 min, and permeablized with 0.1% Triton X-100 in 1xPBS for 30 min at room temperature. ToPro3 was diluted 1:500 in PBS and incubated for 4 hours at room temperature, then washed three times with 1xPBS. The number of cells was estimated by scanning samples on an infrared (IR) laser scanner (Odyssey Licor Biosciences) to quantify integrated infrared intensity of ToPro3. The IR signal was calibrated by quantifying intensity values from different cell densities for 2D and 3D samples prior to perform proliferation assay (Figure S1).

### Immunofluorescent staining

The samples were fixed in 4% paraformaldehyde in 1xPBS for 30 min at room temperature and, after 3 washes with 1xPBS, the cells were permeablized with 0.1% Triton X-100 in 1xPBS for 30 min at room temperature. For filamentous actin staining, phalloidin solution (1:50, Alexa Fluor 594 phalloidin, Invitrogen) was added, incubated at 4 °C for overnight, and washed 3 times with PBS.

### Imaging and analysis

Brightfield images were acquired on an inverted microscope (Eclipse Ti-U, Nikon) using the NIS-Element imaging system (Diagnostic Instruments, Inc.). F-actin and collagen fibers were imaged by using multiphoton laser scanning microscopy (with second harmonic filter for collagen). All multiphoton laser scanning microscopy (MPLSM) and Second Harmonic Generation (SHG) imaging was done on an optical workstation that was constructed around a Nikon Eclipse TE300. A MaiTai Deepsee Ti: sapphire laser (Spectra Physics, Mountain View, CA) excitation source tuned to 890 nm was utilized to generate both multiphoton excitation and SHG. The beam was focused onto the sample with a Nikon (Mehlville, NY) 20X Super Fluor air-immersion lens (numerical aperture (NA) = 1.2). All SHG imaging was detected from the back-scattered SHG signal with a H7422 GaAsP photomultiplier detector (Hamamatsu, Bridgewater, NJ), and the presence of collagen was confirmed by filtering the emission signal with a 445 nm (narrow-band pass) filter (TFI Technologies, Greenfield, MA) to isolate the SHG signal. Acquisition was performed with WiscScan (http://www.loci.wisc.edu/software/wiscscan), a laser scanning software acquisition package developed at LOCI (Laboratory for Optical and Computational Instrumentation, University of Wisconsin, Madison, WI). The morphology analysis of MCF-DCIS clusters was done by using shape descriptor measurement of ImageJ software for aspect ratio (major axis over minor axis).

### Measurement of diffusion in microfluidic channels

The diffusion profiles in the microfluidic device were visualized using the fluorophore Texas Red bound to Dextran 70K MW (Invitrogen, Cat# D-1830) to obtain a diffusion coefficient closer to those of typical light paracrine signaling proteins. In brief, the devices were loaded with a mixed gel in the center chamber, followed by a either the same mixed gel on the outer channels or with a liquid medium using the protocol previously described. The fluorophore was added to the medium at a concentration of 1 µM. Immediately following the addition of the fluorophore, the devices were placed on the IX81 microscope stage (Olympus) and fluorescent timelapse microscopy was performed every 30 min for 9 hours. Images were retrieved and the intensity profile extracted using the software ImageJ. The diffusion pattern was compared to a numerical simulation performed on COMSOL using the 3D diffusion modeling toolbox. A subset of the device, not including the inlet and outlet ports for simplicity purposes, was modeled in 3D. The maximum mesh size was set to 50 µm, the diffusion coefficient of the gel was set to 10µm^2^/s, that of the liquid to 2.10 µm^2^/s, and the fluorophore concentration was set to 0 in areas devoid of compound and 1 in areas containing the compound. A transient solver was used with solution stored every 15 min for a total time of 9 hours. The concentration profile was evaluated on a horizontal plane 50 µm above the floor of the channel, and heat-map images were exported at the desired times.

### mRNA Transcription Analysis

mRNA was isolated from 2D or 3D cultured cells in 24-well using Dynabeads® mRNA DIRECT^TM^ kit (Invitrogen, Cat# 610.21). Then mRNA was reverse transcribed to cDNA using high capacity cDNA reverse transcription kits from Applied Biosystems (Cat# 4374966). Real-time PCR was performed on StepOne Real-Time PCR System (Applied Biosystem) using TaqMan qPCR master mix (Applied Biosystems) along primer/probe sets from Applied Biosystems for the HGF (Hs00300159_m1), MMP14 (Hs01037009_g1), COX2 (Hs01573471_m1), CXCL12 (Hs00171022_m1), and GAPDH (Hs99999905_m1) used as a housekeeping gene to normalize the total number of molecules in each sample. All PCR products had a denaturing step of 95 °C for 15 s, an annealing/extension step at 60 °C for 1 min for a total of 40 cycles. Quantification of mRNA was calculated using relative standard method. Standards are composed of five 1:10 serial dilutions of the same gene.

### Zymography of MMPs Activity

To determine gelatinolytic and caseinolytic activities in HMF conditioned media, zymography was performed using gelatin and casein zymogram gels (Invitrogen). The assay was conducted by following manufacturer’s protocols. Conditioned media from 2D and 3D cultures of HMF cells were collected at 48 hours culture. After being clarified by centrifugation, samples were mixed with 2xSDS sample buffer (Invitrogen) and then subjected to electrophoresis separation at 100V for 90 min. The gels were soaked in Renature buffer for 30min at RT and equilibrated in Develop buffer for 30 min. Then gels were incubated with Develop buffer overnight at 37°C to allow proteinase digestion of its substrate. Gels were stained using GelCodeTM Blue stain reagent (PIERCE) for 2 hours and then destained by DI water. Proteolytic activities appeared as clear bands of lysis against a blue background of stained gelatin or casein. To verify that the detected gelatinolytic and caseinolytic activities were specifically derived from MMPs, the gels were treated in parallel experiments with developing buffer containing 20mM of EDTA.

### Bead-based ELISA

Six different conditioned media from 2D and 3D cultures of HMFs, MCF-DCIS cells, and blank gels were collected after 48 hours of cultivation as described above. Eight magnetic beads coated with specific capture antibodies were selected from three magnetic bead panels. Two Milliplex^®^_MAP_ kits were purchased from Millipore (Human Adipokine Magnetic Bead Panel 2 (HADK2MAG-61K), Human Cytokine Magnetic Bead Panel (HCYTOMAG-60K)). One Bio-Plex Pro™ kit was purchased from Bio-Rad (TGF-β Standard 3-Plex). The assays were conducted by following manufacturer’s protocols. After sample preparation was completed, 96-well plates were introduced into MagPix^®^ instrument (Luminex Corporation) and data collected with xPONENT software (Luminex Corporation).

### HGF ELISA

Conditioned media from 2D and 3D cultures of HMF cells were collected and clarified as above. Human HGF ELISA kit (Invitrogen) was used to detect HGF in conditioned media. Briefly, 50 µl standard dilutions of recombinant human HGF and experimental conditioned media were dispensed into a 96-well plate coated with anti-HGF. The plate was sealed, incubated at room temperature for 3 hours and washed four times with washing buffer. After addition of 100 µl of biotinylated anti-Hu HGF solution and incubation for 1 hour at RT followed by four washes, 100 µl of Streptavidin-HRP was added and incubated for 30 min at RT. After 4 washes, 100µl of stabilized chromogen was added to the wells and incubated for 30 minutes, followed by addition of 100 µl of Stop solution. The absorbance of each well was read at 450 nm using a SpectraMax Plus Spectrophotometer.

### Statistical Analysis

All data were analyzed using the Student’s t-Test, and statistically significantly different conditions (p< 0.05) were used in the results and discussion.

## Supporting Information

Figure S1 **Loading conditions.** (A) a table showing loading conditions for 2D and 3D samples. 2D_L: 2D low density, 2D_H: 2D high density, 3D_0.8: 3D 0.8mg/ml collagen I concentration, 3D_2.0: 3D 2.0mg/ml collagen I concentration. (B) Bar graphs showing the mRNA expressions of HGF, MMP14, COX2, and CXCL12 at each loading condition. ‡ represents a p value of less than 0.05. (C) Calibration curves showing the correlation between the level of integrated intensity and the total number of HMFs per well in 2D (blue) and 3D (green) conditions. (D) Proliferation curves for HMFs cultured in 2D (blue) and 3D (green) conditions. After 48 hours of culturing, the cell densities of HMFs in 2D and 3D became similar (P_0_=0.02, P_24_=0.05, P_48_=0.26).
(JPG)

Figure S2 **HGF mRNA expressions in MCF-DCIS cells cultured in 2D and 3D conditions.** HGF mRNA was undetectable in MCF-DCIS cells in both 2D and 3D culture conditions. HGF mRNA expression in HMF cells was used as a positive control. ‡ represents a p value of less than 0.05.
(TIFF)

Figure S3 **Description of microfluidic channel dimensions.**
(TIFF)

Figure S4 **Demonstration of channel loading using red and blue food coloring dyes.**
(TIFF)

Figure S5 **Numerical simulation of the diffusion profile in the microdevice containing 3D gel in the center chamber as well as in the outer channels.** (A) A set concentration of fluorophore was placed in the outer channels and allowed to diffuse inward. (B) A set concentration of fluorophore was placed in the inner chamber and allowed to diffuse outward.
(TIFF)

Figure S6 **Averaged aspect ratio of MCF-DCIS clusters and the mean intensity of SHG.** (A) MCF-DCIS cluster (co-cultured with 3D HMF) shape analysis by estimating averaged aspect ratio. Both HGF neutralizing antibody and c-met inhibitor (anti c-met) decreased the aspect ratio of MCF-DCIS clusters and the mean intensity of SHG in 3D/3D co-culture. ‡ represents a p value of less than 0.05. (B) The average aspect ratio of MCF-DCIS clusters and the mean intensity of SHG with 2D HMF.
(TIFF)

Figure S7 **The effect of β1 integrin function blocking antibody.** Bar graph shows data from HGF ELISA performed with conditioned media collected from 2D and 3D cultures of HMF and also with the β1 integrin function blocking antibody (25 μg/ml). ‡ represents a p value of less than 0.05.
(TIFF)

Table S1 **Summary of the differences of macro (i.e., transwells) vs. micro co-culture systems.**
(PDF)

Movie S1 **A movie showing loading process into a three compartments microfluidic channel with red and blue food coloring dyes.**
(MOV)
