# Supplementary figures and images for: Understanding the Impact of 2D and 3D Fibroblast Cultures on In Vitro Breast Cancer Models
Source: PLoS One. 2013 Oct 4;8(10):e76373. doi: 10.1371/journal.pone.0076373 (PMC3790689; doi:10.1371/journal.pone.0076373)

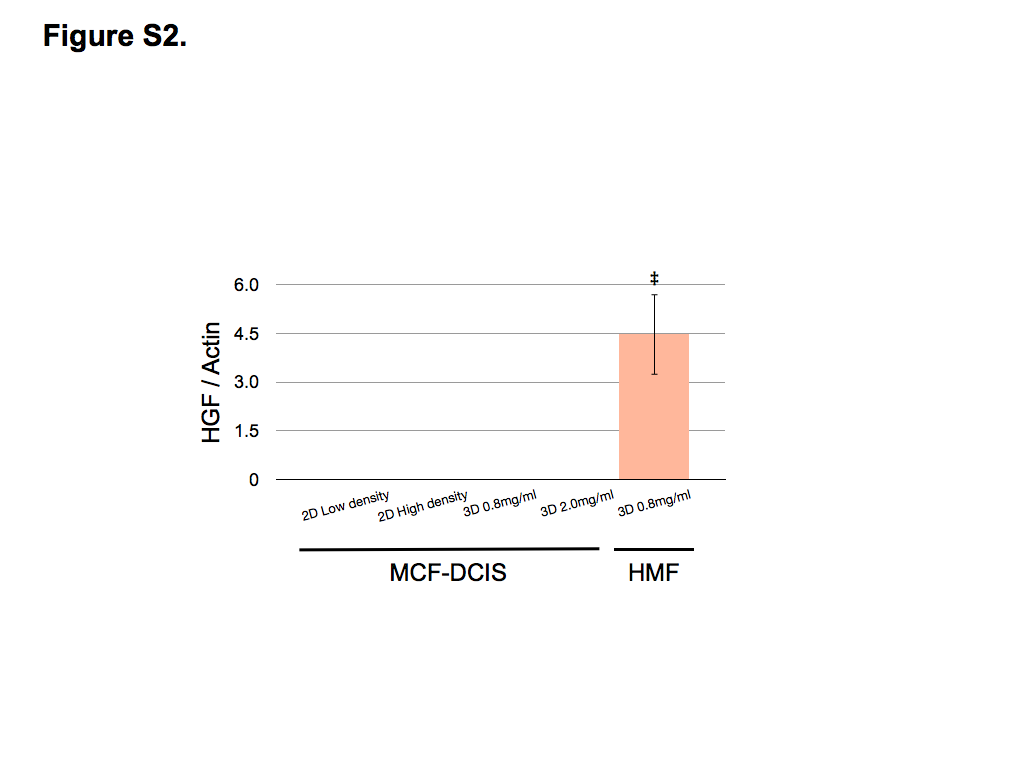

Supplement: Figure S2 — HGF mRNA expressions in MCF-DCIS cells cultured in 2D and 3D conditions. HGF mRNA was undetectable in MCF-DCIS cells in both 2D and 3D culture conditions. HGF mRNA expression in HMF cells was used as a positive control. ‡ represents a p value of less than 0.05. (TIFF) [file pone.0076373.s002.tiff]

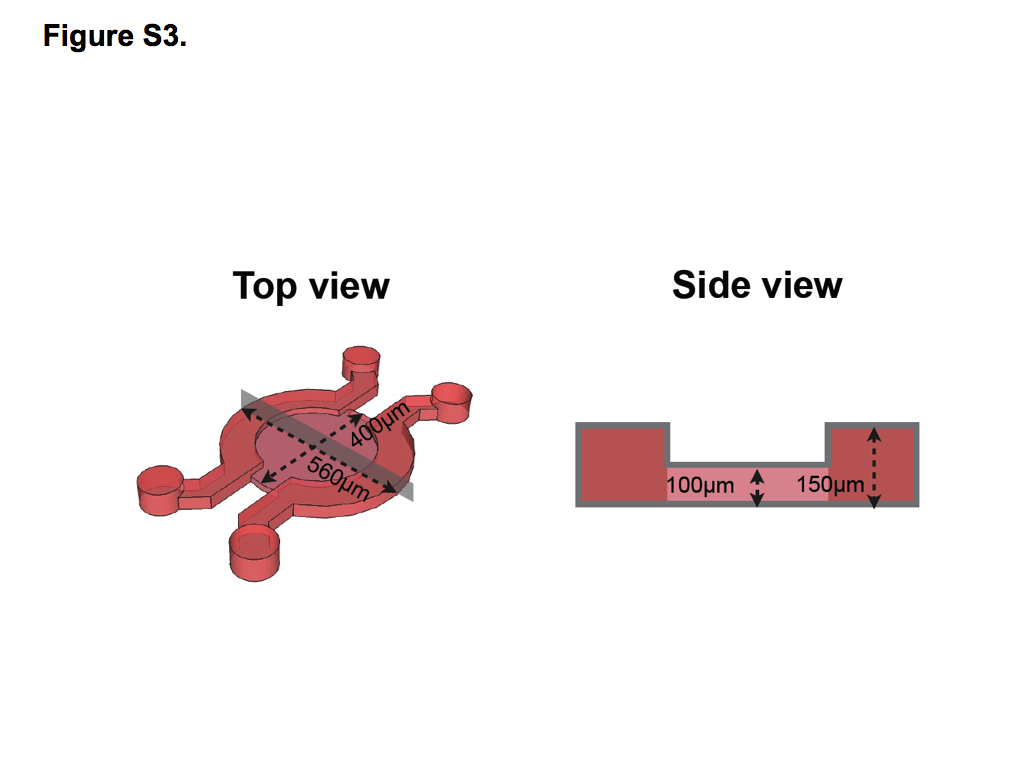

Supplement: Figure S3 — Description of microfluidic channel dimensions. (TIFF) [file pone.0076373.s003.tiff]

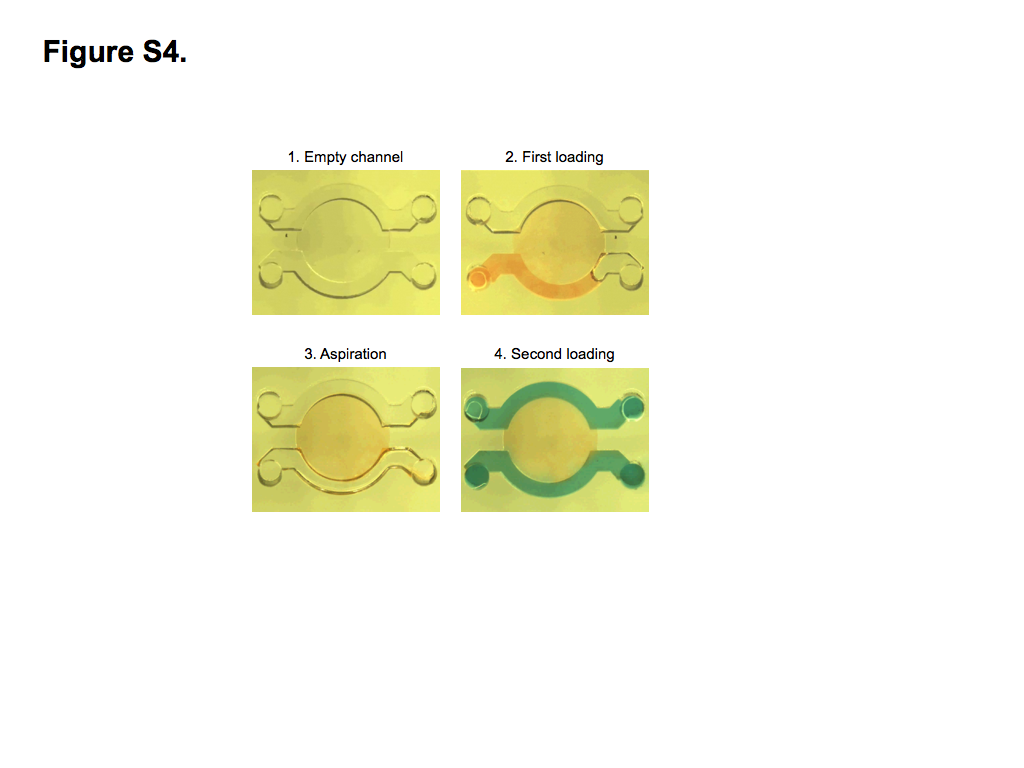

Supplement: Figure S4 — Demonstration of channel loading using red and blue food coloring dyes. (TIFF) [file pone.0076373.s004.tiff]

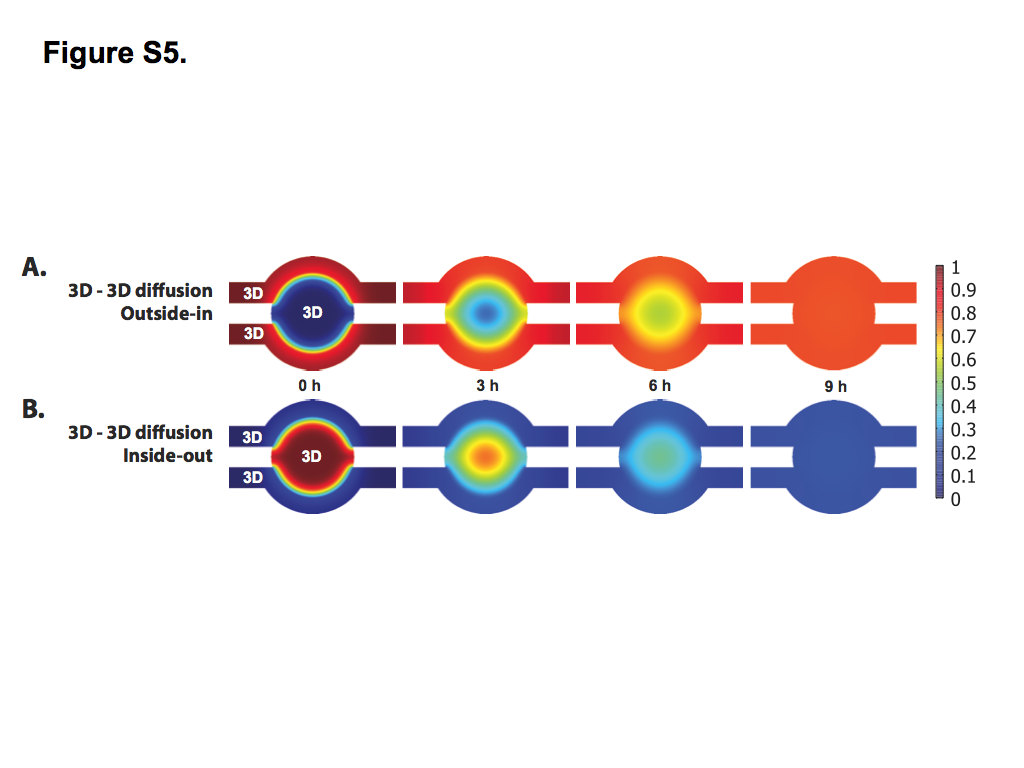

Supplement: Figure S5 — Numerical simulation of the diffusion profile in the microdevice containing 3D gel in the center chamber as well as in the outer channels. (A) A set concentration of fluorophore was placed in the outer channels and allowed to diffuse inward. (B) A set concentration of fluorophore was placed in the inner chamber and allowed to diffuse outward. (TIFF) [file pone.0076373.s005.tiff]

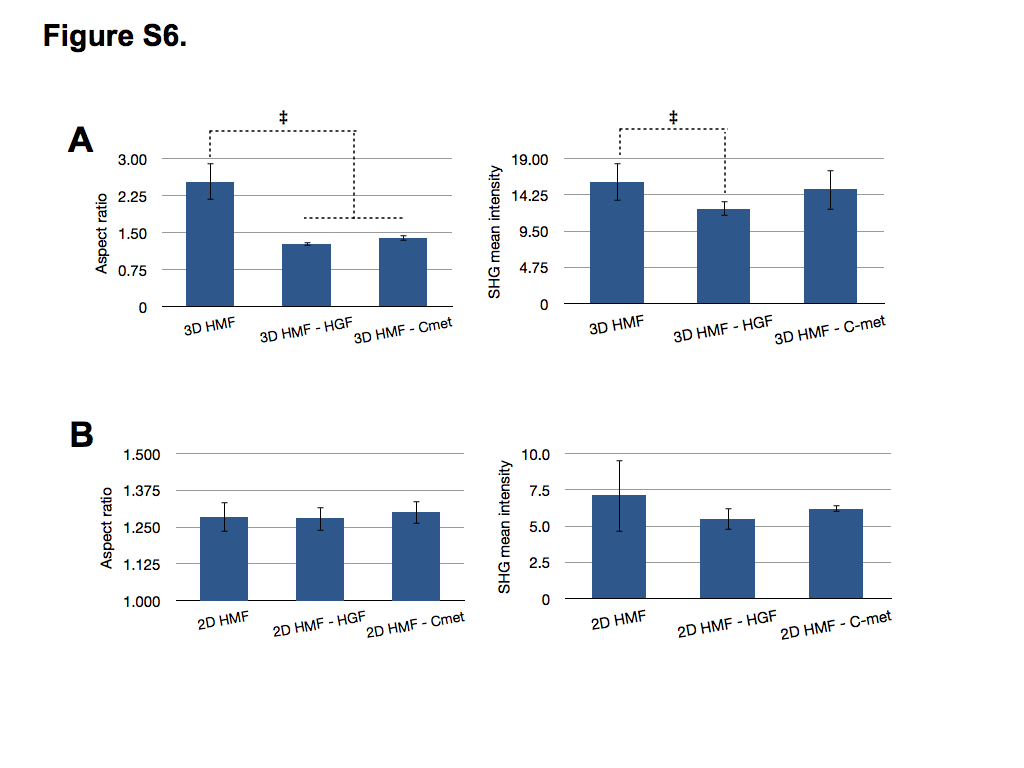

Supplement: Figure S6 — Averaged aspect ratio of MCF-DCIS clusters and the mean intensity of SHG. (A) MCF-DCIS cluster (co-cultured with 3D HMF) shape analysis by estimating averaged aspect ratio. Both HGF neutralizing antibody and c-met inhibitor (anti c-met) decreased the aspect ratio of MCF-DCIS clusters and the mean intensity of SHG in 3D/3D co-culture. ‡ represents a p value of less than 0.05. (B) The average aspect ratio of MCF-DCIS clusters and the mean intensity of SHG with 2D HMF. (TIFF) [file pone.0076373.s006.tiff]

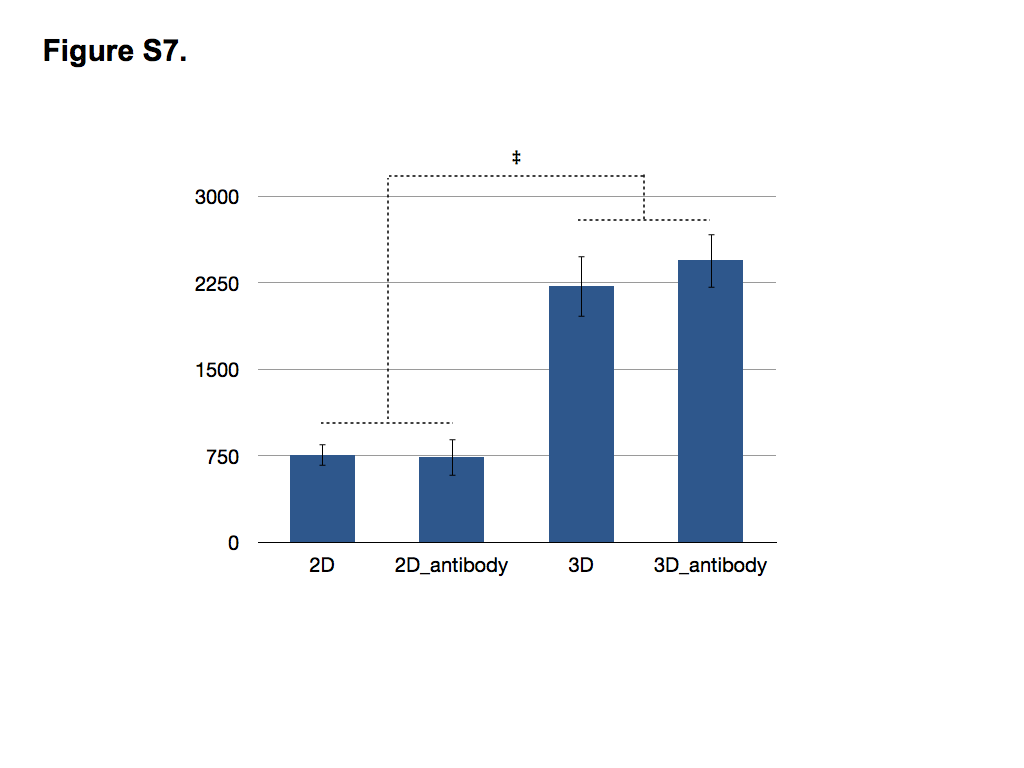

Supplement: Figure S7 — The effect of β1 integrin function blocking antibody. Bar graph shows data from HGF ELISA performed with conditioned media collected from 2D and 3D cultures of HMF and also with the β1 integrin function blocking antibody (25 μg/ml). ‡ represents a p value of less than 0.05. (TIFF) [file pone.0076373.s007.tiff]
