# Supplementary material for: Understanding the Impact of 2D and 3D Fibroblast Cultures on In Vitro Breast Cancer Models
Source: PLoS One. 2013 Oct 4;8(10):e76373. doi: 10.1371/journal.pone.0076373 (PMC3790689; doi:10.1371/journal.pone.0076373)
Supplement: Table S1 — Summary of the differences of macro (i.e., transwells) vs. micro co-culture systems. (PDF) [file pone.0076373.s008.pdf]

Supplementary Table 1.

	Transwell (24 wells)	Microsystem
Surface area	• Insert: 0.3 cm^2<br>• Bottom well: 2.0 cm^2	• Center chamber: 12.56 mm^2<br>• Side chambers:12.05 mm^2
Volume	• Insert: 0.2 ml<br>• Bottom well: 0.7 ml	• Center chamber: 1.25 μl<br>• Side chambers: 3.69 μl
